# Quantile regression to examine the association of air pollution with subclinical atherosclerosis in an adolescent population

**DOI:** 10.1016/j.envint.2022.107285

**Published:** 2022-05-10

**Authors:** Adjani A. Peralta, Joel Schwartz, Diane R. Gold, Judith M. Vonk, Roel Vermeulen, Ulrike Gehring

**Affiliations:** a Department of Environmental Health, Harvard T.H. Chan School of Public Health, United States; b Department of Epidemiology, Harvard T.H. Chan School of Public Health, United States; c Channing Division of Network Medicine, Department of Medicine, Brigham and Women’s Hospital and Harvard Medical School, United States; d Department of Epidemiology and Groningen Research Institute for Asthma and COPD, University of Groningen, University Medical Center Groningen, The Netherlands; e Institute for Risk Assessment Sciences, Utrecht University, The Netherlands

**Keywords:** Quantile regression, Environmental Epidemiology, Air pollution, Cardiovascular disease, Atherosclerosis, Adolescents

## Abstract

**Background:**

Air pollution has been associated with carotid intima-media thickness test (CIMT), a marker of subclinical atherosclerosis. To our knowledge, this is the first study to report an association between ambient air pollution and CIMT in a younger adolescent population.

**Objective:**

To investigate the associations beyond standard mean regression by using quantile regression to explore if associations occur at different percentiles of the CIMT distribution.

**Methods:**

We measured CIMT cross-sectionally at the age of 16 years in 363 adolescents participating in the Dutch PIAMA birth cohort. We fit separate quantile regressions to examine whether the associations of annual averages of nitrogen dioxide (NO_2_), fine particulate matter (PM_2.5_), PM_2.5_ absorbance (a marker for black carbon), PM_coarse_ and ultrafine particles up to age 14 assigned at residential addresses with CIMT varied across deciles of CIMT. False discovery rate corrections (FDR, p < 0.05 for statistical significance) were applied for multiple comparisons. We report quantile regression coefficients that correspond to an average change in CIMT (μm) associated with an interquartile range increase in the exposure.

**Results:**

PM_2.5_ absorbance exposure at birth was statistically significantly (FDR < 0.05) associated with a 6.23 μm (95% CI: 0.15, 12.3) higher CIMT per IQR increment in PM_2.5_ absorbance in the 10th quantile of CIMT but was not significantly related to other deciles within the CIMT distribution. For NO_2_ exposure we found similar effect sizes to PM_2.5_ absorbance, but with much wider confidence intervals. PM_2.5_ exposure was weakly positively associated with CIMT while PM_coarse_ and ultrafine did not display any consistent patterns.

**Conclusions:**

Early childhood exposure to ambient air pollution was suggestively associated with the CIMT distribution during adolescence. Since CIMT increases with age, mitigation strategies to reduce traffic-related air pollution early in life could possibly delay atherosclerosis and subsequently CVD development later in life.

## 1. Introduction

Cardiovascular disease (CVD) accounts for the world’s leading cause of both mortality and morbidity in developing and non-developing nations (Kaptoge et al., 2019; McAloon et al., 2016). Specifically, exposure to air pollution each year accounts for approximately seven million premature deaths worldwide (Organization, 2014). The acceleration of atherosclerosis, a collective term for fibrous plaques occurring in the innermost layer of arteries, has been suggested as one of the underlying biological mechanisms that connects long-term air pollution exposure to CVD (Yang et al., 2017; Brook et al., 2010).

Cumulative exposure to higher levels of inhaled air pollution can promote the presence and intensity of atherosclerotic lesions through higher systemic inflammation and oxidative stress (Ho et al., 2013; Kattoor et al., 2017). An individual’s cumulative exposure to several risk factors such as genetic susceptibility, obesity, smoking behavior and other lifestyle factors, can contribute to the progression of atherosclerosis (Urbina et al., 2008; Chambless et al., 2002). Long-term effects of air pollution exposure on higher carotid intima-media thickness (CIMT), a measure of subclinical atherosclerosis (Hodis et al., 1998), have been documented in adults (Künzli et al., 2005; Wilker et al., 2013; Diez Roux et al., 2008), but no studies have shown an association in younger adolescent populations (Lenters et al., 2010; Breton et al., 2012; Iannuzzi et al., 2010). Since atherosclerosis starts in childhood with deposits of cholesterol inside arteries (Origin of atherosclerosis in childhood and adolescence, 2000), we hypothesize that air pollution exposure during the prenatal period and early childhood may be associated with early signs of subclinical atherosclerosis in younger adolescence.

Previously, adolescent studies (Lenters et al., 2010; Breton et al., 2012; Iannuzzi et al., 2010; Breton et al., 2016) have used standard regression techniques that report the change in the mean value of CIMT for a given change in air pollution exposure. Studying the effect of air pollution on CIMT by calculating averages may not capture the heterogeneity in the influence of air pollution during early adolescence. Thus, we hypothesize that mean regression analyses could miss associations that transpire in the tails of the outcome distribution. Quantile regression can estimate associations with air pollution at various percentiles of the outcome distribution (Lê Cook and Manning, 2013). This technique permits one to distinguish if certain parts of the distribution of CIMT are more affected by the exposure.

To address our hypothesis, we utilized quantile regression to investigate the association between childhood exposure up to the age of 14 years to NO_2_, PM_2.5_, PM_2.5_ absorbance, PM_coarse_ and ultrafine particles and CIMT within deciles of the CIMT distribution in a population of 16-year-old adolescents participating in the Prevention and Incidence of Asthma and Mite Allergy (PIAMA) birth cohort in the Netherlands. Previously, no study has reported associations between air pollution and CIMT in the PIAMA cohort.

## 2. Methods

### 2.1. Study population

Participants included in this study were part of the Prevention and Incidence of Asthma and Mite Allergy (PIAMA) population-based birth cohort established in the Netherlands in 1996. Details of the recruitment and design of the cohort have been described previously in depth (Brunekreef et al., 2002; Gehring et al., 2011). Mothers were recruited during their second trimester of pregnancy at various prenatal clinics in the North, West and Central regions of the Netherlands initially for the purposes of studying asthma and allergies. In total, 3963 children born between 1996 and 1997 were recruited for the baseline study population. Questionnaires were sent to the children’s parents during pregnancy and at regular intervals afterwards (3 months after birth and annually starting at 1 year of age until age 8 and then at ages 11, 14). Starting from 11 years of age, the children were also provided with their own questionnaires to fill out. The participants were also invited for medical examinations at the age of 1, 4, 8, 12 and 16 years. CIMT measurements were performed at the age of 16 at the Utrecht study center.

From the 1232 PIAMA participants that were invited, 437 participated in the IMT measurements and 420 had a valid measurement for CIMT (Prins-van Ginkel et al., 2018). After merging the participant information with the air pollution exposures, 398 participants remained in the analysis. Sixteen individuals had missing information on whether their mother was considered overweight before pregnancy and two had missing information on parental education. Seven participants had missing information on their systolic blood pressure measurement and ten on their nationality leaving 363 participants for the final analysis.

For some participants, residential histories are incomplete due to reporting inaccuracies (non-valid addresses or addresses outside of the Netherlands) while for others, the study did not capture a participant’s moving date, so exposure levels for a specific time point could not be assigned. However, the level of missingness for the air pollution exposures was < 0.06% for various concentrations of air pollutants and depended on the specific timeframe.

### 2.2. Annual air pollution concentrations

Annual average air pollution concentrations at the participant’s residential addresses were estimated with previously validated land-use regression (LUR) models originating from the ESCAPE project (Beelen et al., 2013; Eeftens et al., 2012). Details and the development of LUR models have been described previously (Beelen et al., 2013; Eeftens et al., 2012). Briefly, air pollution monitoring data was collected during a year-long campaign that started in February 2009. NO_2_ measurements were conducted at 80 sites for intervals of two weeks, three times in the different seasons (cold, warm, and intermediate). Measurements of particulate matter (PM_2.5_, PM absorbance and PM_coarse_) were conducted at the same time for half the sites. The three measurement periods were averaged to produce annual averages for pollutant per location. Predictors included population density, traffic intensity and land use variables to account for spatial variation (Beelen et al., 2013; Eeftens et al., 2012). The spatial LUR models were used to estimate annual averages of air pollutants from birth to age 14 for NO_2_, PM_2.5_, PM_2.5_ absorbance and PM_coarse_ assigned at the participants’ residential addresses on their birthdays. We did not consider changes in address between the participants’ birthdays without back-extrapolation, i.e. changes in exposure are entirely due to changes in address and do not account for temporal trends. Most of the exposure models performed well with R^2^ ranging between 61 and 89% for leave one out cross-validation, but the R^2^ for PM_coarse_ was lower at 38% (see Supplementary 1).

Annual average ultrafine particle concentrations at the participant’s residential addresses were estimated from a national spatial model that integrated both regional background measurements and mobile monitoring at several road segments (Kerckhoffs et al., 2021). Briefly, regional background ultrafine particle concentrations were collected across 20 regional background sites at various locations in the Netherlands three times each for two weeks. The annual average ultrafine background concentrations were estimated with a kriging method (van de Beek et al., 2021). In addition, mobile monitoring of ultrafine particle concentrations was conducted between June 2016 to November 2017 with an electric car (REVA, Mahindra Reva Electric Vehicles Pvt. Ltd., Bangalore, India) on 14,393 road segments between 9:15am and 4:00 pm. By limiting the hours in which monitoring took place, the measurements improved comparability between the different road segments by avoiding high road traffic periods (Kerckhoffs et al., 2016; van Nunen et al., 2017). A condensation particle counter (TSI, CPC 3007) calculated the ultrafine particle concentrations in the back of the car and any repeated road segments were averaged together.

### 2.3. Carotid intima-media thickness test (CIMT)

During the 16-year medical examination, trained medical technicians used a portable ultrasound system, the Panasonic CardioHealth Station (Panasonic Healthcare), to automatically measure bilaterally the IMT in the distal common carotid artery proximal to the bifurcation at six standard angles (left: 210°, 240° and 270°; right: 90°, 120° and 150°). The CardioHealth Station minimizes measurement error by automatically identifying the relevant region and measures CIMT over a standard length of 10 mm in the end-diastolic phase of a participant’s heartbeat. The mean CIMT measurement utilized as the primary outcome in this analysis was determined by averaging the six IMT measurement angles. If less than six measurements were available, the mean CIMT was computed with the available measurements if there were at least 3 IMT measurements available.

### 2.4. Covariates

Questionaries completed by the parents provided the relevant information on potential confounders along with data collected at the 16-year-old medical examination. We considered two models. The first model was adjusted for covariates but excluded cardiometabolic variables (body mass index (BMI) (kg/m^2^), serum total cholesterol (TC) and high-density lipoprotein cholesterol (HDLC) and mean systolic and diastolic pressure at the medical exam) that might be on the casual pathway and the final main model adjusted for all potential confounders.

We adjusted for the following potential confounders: age, sex, BMI (kg/m^2^) at the medical exam, TC and HDLC (mmol/L) at the medical exam (Berentzen et al., 2014), mean systolic and diastolic pressure at the medical exam (mm Hg) (Bilenko et al., 2015), parental education (high = higher vocational education or university studies), exposure to indoor tobacco smoke, maternal smoking status during pregnancy, maternal BMI (kg/m^2^), breastfeeding status and Dutch nationality (both parents born in the Netherlands). These potential confounders were selected a priori based on previous literature (Prins-van Ginkel et al., 2018; Gall et al., 20142014; Caserta et al., 2010). Exposure to indoor smoke, a binary variable, corresponds to if any household members smoked inside the residential home at least once a week within the exposure period (Lakwijk et al., 1998).

During the medical exam at age 16, a blood sample was collected where both TC and HDLC were calculated enzymatically utilizing the Roche automated clinical chemistry analyzers (Roche Diagnostics, Indianapolis, IN, USA) (Berentzen et al., 2014).

### 2.5. Statistical Methods

We fit squantile regressions to examine the associations between the annual averages of air pollutants up to age 14 for nitrogen dioxide (NO_2_), fine particulate matter (PM_2.5_), PM_2.5_ absorbance (a marker for black carbon) and PM_coarse_ assigned at the participants’ residential addresses and CIMT within deciles of the CIMT distribution (10th to 90th deciles). By using the fully adjusted model as the main model, the reported estimates only account for the direct effects of the exposure on CIMT and remove any effects that are mediated by the other variables. Thus, we present conservative effect estimates for the association between air pollutants and CIMT within the distribution of CIMT.

Quantile regression makes no assumptions about the distribution of the residuals and allows us to explore the associations outside of the mean of CIMT (Waldmann, 2018). We report the quantile regression coefficients that corresponded to an average difference in CIMT (μm) associated with an interquartile range (IQR) difference in the annual air pollution concentrations and 95% bootstrap confidence intervals. The confidence intervals were constructed with the *quantreg* package (Koenker and quantreg, 2009) using the xy-pair bootstrapping method with 200 bootstrapping replications. (Koenker, 1994) We utilized IQR since it indicates the spread of the exposure distribution (25th to 75th percentiles) in our predicted data and facilitates comparison of associations between pollutants. False discovery rate corrections (FDR, p < 0.05 for statistical significance) (Benjamini and Hochberg, 1995) were applied for multiple comparisons across the different exposures and deciles of CIMT. The corrected FDR *p*-values were calculated with the p. adjust function in base R using the Benjamini and Hochberg correction (Benjamini and Hochberg, 1995).

All statistical analyses were conducted using R version 4.0.5 (R Foundation for Statistical Computing, Vienna, Austria) and the *quantreg* package.

## 3. Results

Table 1 shows the characteristics of the 363 adolescents included in the study population. The study population in comparison to the PIAMA baseline population had a higher percentage of participants with highly educated parents and participants who received breastfeeding for more than 16 weeks and lower percentages of participants with exposure to indoor smoke (Supplementary 2). The average CIMT measurement was 468 μm (SD 39.2 μm) at the mean age of 16.3 years.

Table 2 shows the distribution of the annual average air pollution concentrations for NO_2_, PM_2.5_, PM_2.5_ absorbance, PM_coarse_ and ultrafine particles assigned at the participants’ residential birth addresses in the PIAMA cohort. The average CIMT measurement was 468 μm (SD 39.2 μm) at the mean age of 16.3 years. Supplementary 3 provides the distribution of CIMT measured at the age of 16 years for the PIAMA participants. While Supplementary 4 shows the distribution of annual average air pollutant concentrations at the birth address for NO_2_, PM_2.5_, PM_2.5_ absorbance and PM_coarse_ and CIMT within deciles of the CIMT distribution. The average CIMT measurement was 405 μm (SD 12.2 μm) within the 10th decile of the CIMT distribution and 544 μm (SD 23.9 μm) within the 90th decile of the CIMT distribution.

The estimates of IQR used as exposure increments in this analysis can be found in Table 2. The median concentrations for the air pollutants were 24.55 μg/m^3^ for NO_2_, 16.65 μg/m^3^ for PM_2.5_, 1.29 10^−5^m^−1^ for PM_2.5_ absorbance, 8.28 μg/m^3^ for PM coarse and 1.10 particles/10,000*cm^3^ for ultrafine particles. The air pollutants were positively correlated with one another with the highest correlation between NO_2_ and ultrafine particles (Spearman correlation coefficient, ρ = 0.81). Across time periods of the same air pollutant, exposure periods were highly correlated with one another (see Supplementary 2). For example, the 3rd and 4th year average of NO_2_ had a Spearman correlation coefficient of ρ = 0.94. On average, the annual average levels of air pollutants were lower at 14 years compared to 0 years (NO_2_ (2.08%), PM_2.5_ absorbance (2.33%), PM_coarse_ (0.85%) and ultrafine particles (3.08%)) while PM_2.5_ increased by 0.54%. These changes are due to residential mobility since the exposure models only account for spatial not temporal trends (Beelen et al., 2013; Eeftens et al., 2012).

Across the different associations of annual averages of air pollutants up to the age of 14 years for NO_2_, PM_2.5_, PM_2.5_ absorbance, PM_coarse_ and ultrafine particles assigned at the participants’ residential addresses, we found that the strongest statistically significant association was reported with the estimated exposure at the birth address (Supplementary 6, Table 3). We found suggestions that exposure to higher levels of air pollution during earlier years was associated with a higher CIMT especially at the lower tails of the CIMT distribution while exposure levels in later years were not (Supplementary 6).

PM_2.5_ absorbance exposure at birth was the only statistically significant association (false discovery rate (FDR) < 0.05) for both Model I and Model II (see Table 3). The main model that adjusted for all potential confounders found a 6.23 μm (95% CI: 0.15, 12.30) higher CIMT per IQR increment in PM_2.5_ absorbance for the 10th quantile of CIMT but was not significantly related to the 50th quantile (estimate: 1.69 μm (95% CI: −5.59, 8.97) nor the 90th quantile (estimate: −4.54 μm (95% CI: −12.11, 3.03). While IQR increments in NO_2_, PM_2.5_, PM_coarse_ and ultrafine particles were not associated with significantly higher CIMT, PM_2.5_ exposure at birth was weakly associated (*p*-value < 0.10) with higher CIMT at the 10th quantile (estimate: 4.85 μm (95% CI: −0.41, 10.10) and a 3.73 μm (95% CI: −0.74, 8.20) increase per IQR for the 20th quantile of CIMT. For NO_2_ exposure we found similar association estimates as for PM_2.5_ absorbance, but with much wider confidence intervals (NO_2_ exposure at birth estimate at the 10th quantile of CIMT: 7.11 μm (95% CI: −17.1, 31.32)). PM_coarse_ and ultrafine particles did not report any statistically significant associations nor patterns across any of the quantiles of CIMT.

**Fig. 1.**
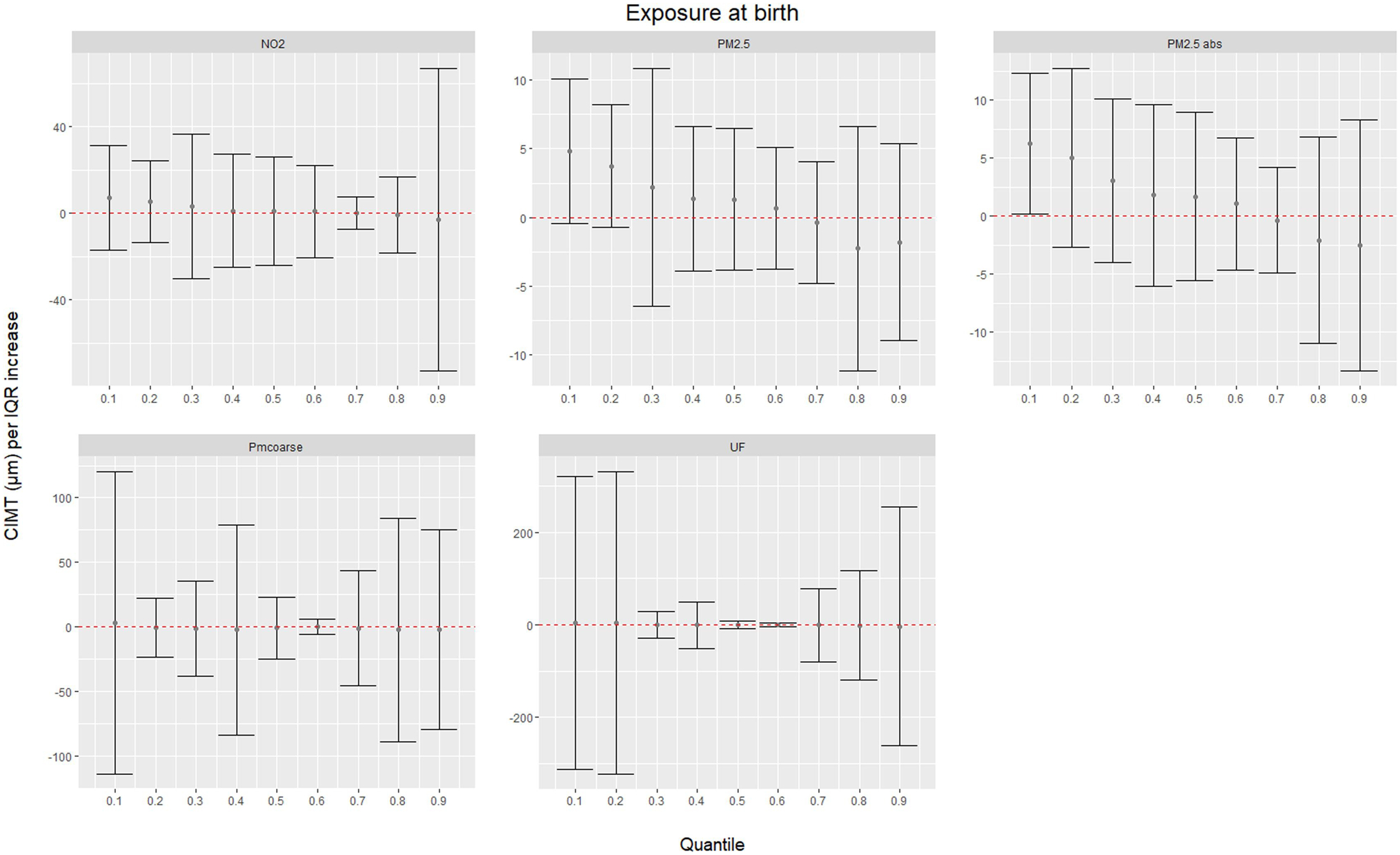
Associations between air pollutants at birth and quantiles of the distribution of common carotid intima media thickness (CIMT) measured at age 16 years (adjusted for age, sex, parental education (high = vocational education or university studies), exposure to indoor tobacco smoke, maternal smoking status during pregnancy, maternal BMI (kg/m2), breastfeeding status, Dutch nationality (both parents born in the Netherlands), body mass index (BMI) (kg/m2), TC and HDLC (mmol/L) and mean systolic and diastolic pressure at the medical exam (mm Hg)). The y-axes represent the difference in CIMT (μm) for an IQR increment in exposure. The error bars represent 95% bootstrap confidence intervals.

**Fig. 2.**
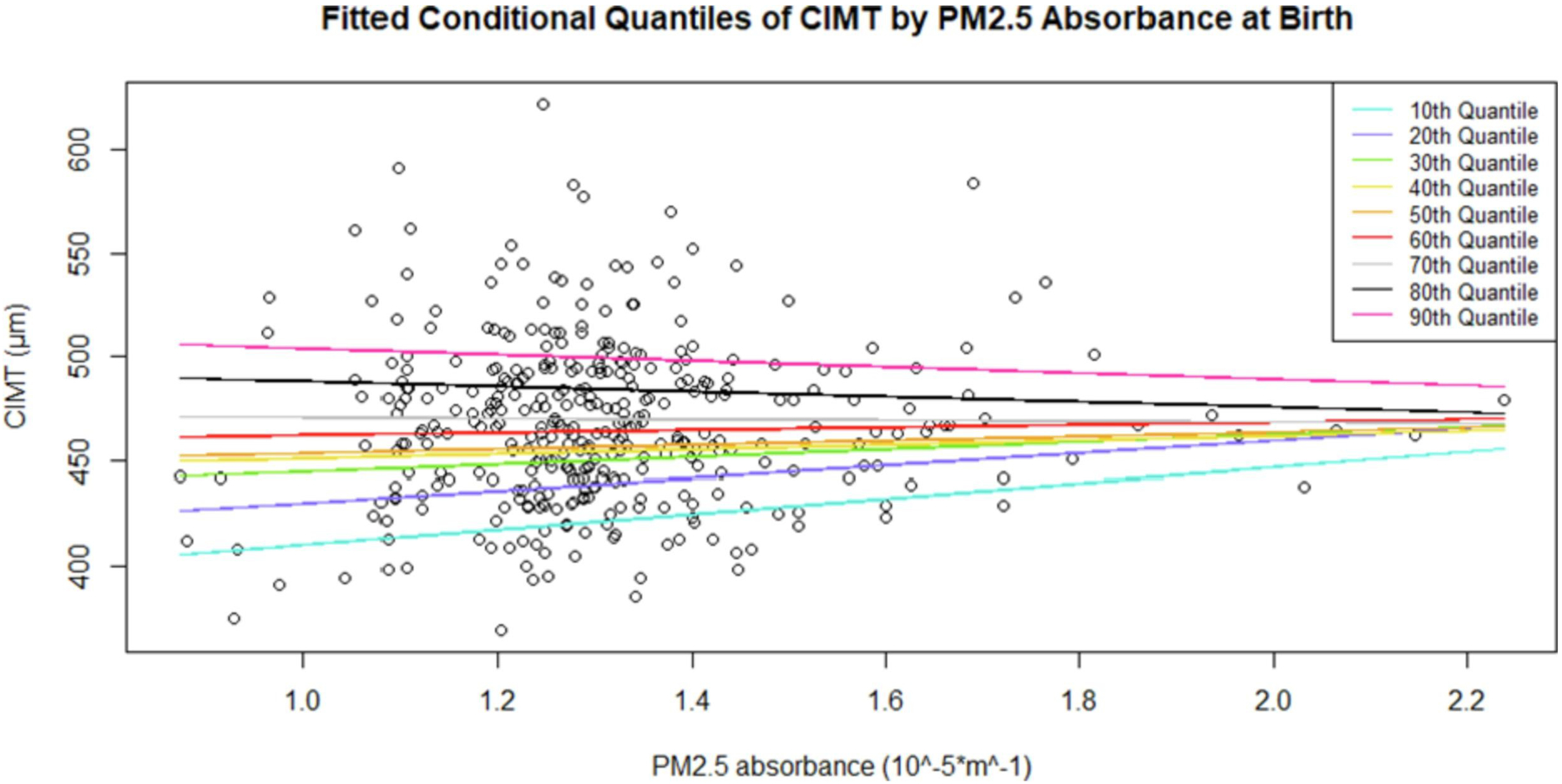
Predicted 10th to 90th quantiles of common carotid intima media thickness (CIMT) conditional on PM_2.5_ absorbance at birth address in the PIAMA cohort.

Fig. 1 illustrates associations between air pollutants at birth and quantiles of the distribution of CIMT in the fully adjusted model. The figure highlights that the association estimate of PM_2.5_ absorbance and to a lesser extent PM_2.5_ and NO_2_ exposure starts to decrease across increasing deciles of CIMT. PM_coarse_ and ultrafine particles do not display a clear pattern across deciles of CIMT. Fig. 2 shows the predicted 10th to 90th quantiles of CIMT conditional on PM_2.5_ absorbance at birth in the fully adjusted model.

## 4. Discussion

Our study adds to the sparse literature examining the association between early life exposure to air pollution and the possible risk of subclinical atherosclerosis among adolescents. We found that among 16-year-old PIAMA participants, higher levels of PM_2.5_ absorbance, a marker for black carbon, was associated with higher levels of CIMT at the 10th quantile while we found no statistically significant associations for NO_2_, PM_2.5_, PM coarse and ultrafine particles after adjustment for multiple testing. We also found a positive trend with annual PM_2.5_ and NO_2_ exposure at birth and elevated CIMT levels for the lower tails of the outcome distribution. Our results suggest higher levels of that early life traffic-related air pollution exposure, at birth, could possibly increase an adolescent’s risk for heart disease in the future.

Previous studies in adults have reported associations between long-term air pollution and (Perez et al., 2015), but limited research exists on young adolescent populations. The few reported studies in young children or adolescents have focused on two cardiovascular markers of subclinical atherogenesis: carotid artery arterial stiffness (CAS) and carotid artery intima-media thickness (CIMT). While these studies report an association between certain air pollutants and CAS, none have found significant associations between NO_2_, PM_2.5_, PM_2.5_ absorbance or PM_coarse_ and CIMT. A possible reason is that CIMT requires a structural change in the arteries that in comparison to CAS takes longer to develop into discernable differences. For example, Breton et al. found that among college students with an average age of 20 years, a 2 SD increase in prenatal PM_2.5_ exposure was associated with different CAS indices (Carotid stiffness index beta: +5% (95% CI: 0–10%, Young’s elastic modulus: +5% (95% CI: 1–10%) and distensibility: −5% (95% CI: −9 to 1%)) (Breton et al., 2016). However, no associations were found between any pre- or postnatal exposure to air pollutants and CIMT. Iannuzzi et al. reported that children (6–14 years of age) living closer to a main road had higher indexes of CAS than children living further away, but no associations were found with CIMT (Iannuzzi et al., 2010). Lenters et al. found that among young adults with an average age of 28.4 years, a 25 μg/m^3^ increase in NO_2_ exposure was associated with CAS indexes (4.1%, 95% CI: 0.1 to 8%, increase in pulse wave velocity and 37.6%, 95% CI: 2.2 to 72.9%, increase in augmentation index), but no associations were found with CIMT (Lenters et al., 2010). While we observe that PM_2.5_ absorbance, a marker for black carbon, exposure at birth was associated with CIMT at the 10th quantile, the association was found only at the lowest tail of the CIMT distribution. This is the first study to report a possible association between the lowest decile of CIMT and traffic-related air pollutant among an adolescent population. Further studies are needed to explore if the association between PM_2.5_ absorbance and CIMT can be found at other quantiles of the CIMT distribution for other adolescent populations.

We only found associations at the lowest decile of CIMT, which are the adolescents at the lowest risk for carotid atherosclerotic vascular disease. While an effect in the lowest decile of CIMT could have a lower immediate impact than if it was in the highest decile, this increase during adolescence could subsequently lead to a higher CIMT at a younger age than they could otherwise experience. Since many studies have documented that CIMT increases with age (Sass et al., 1998; Youn et al., 2011; Engelen et al., 2013), raising CIMT in younger populations may possibly increase susceptibility to CVD earlier in their life course.

In the Bogalusa Heart study, a longitudinal cohort of young adults based in Louisiana, Stein et al. reported average estimates of CIMT by age, sex and race (Stein et al., 2004). Specifically, among 25-year-old participants in the 10th percentile of CIMT, the average composite CIMT was 576 μm for white males, 788 μm for black males, 575 μm for white females and 612 μm for black females. Within the same cohort, Johnson et al. reported that the average composite CIMT among young adults between the ages of 25 to 37 years increased by 17 ± 26 μm per year (Johnson et al., 2007). In addition, the Atherosclerosis Risk in Young Adults study with an average age of 28.4 years based in Utrecht, The Netherlands found the mean CIMT to be 490 μm (Lenters et al., 2010). In comparison, the average CIMT was lower at 405 μm in the 10th percentile of the CIMT distribution among our adolescent population. Both the Bogalusa Heart and Atherosclerosis Risk in Young Adults studies report a higher average CIMT among their older participants. It is possible that the Bogalusa Heart study reports an overall higher CIMT in comparison to the Atherosclerosis Risk in Young Adults study due to differences in generic and environmental backgrounds.

The fetal origins hypothesis suggests that adverse nutrition during the prenatal period can increase an individual’s susceptibility to atherosclerosis-related diseases and CVD in later adulthood (Barker et al., 1989; Martyn et al., 1998). Studies have shown that distinct windows of susceptibility during the prenatal and infancy period can impact one’s overall adult health (Cohen Hubal et al., 2008). Both epidemiological and animal models have shown that early life exposure to air pollution can later increase susceptibility towards adult CVD (Gorr et al., 2014; Weldy et al., 2014; Painter et al., 2005).

While this study finds an association between prenatal exposure to traffic particles and CIMT at the lowest end of the CIMT distribution, additional studies are needed in other adolescent populations with longitudinal measurements.

## 5. Strengths and limitations

A major strength of our study is that it focuses on the first 16-year-old adolescent population and utilizes quantile regression as a novel method to explore the association between air pollution and CIMT. Quantile regression allows us to find associations for the overall shape of the outcome distribution, as opposed to only the mean (Lê Cook and Manning, 2013). Quantile regression does not make assumptions of the distribution of the residuals or the outcome in contrast to ordinary least squares (OLS) utilized by the typical linear regressions used to investigate these associations. OLS assumes that the associations between the dependent and independent variables are the same at all levels. Quantile regression allows us to explore associations at the tails of the distribution that are potentially missed by OLS since it only focuses on the mean. Other strengths include the extensive collection of information since the start of the birth cohort on a range of potential confounders and the study also accounted for multiple testing by correcting with the FDR.

Our study does include limitations. Our findings cannot be used for causal inference due to the cross-sectional nature of only having one measurement of CIMT for our participants. In the future, longitudinal studies are needed to explore if these associations continue in a younger adult population or can be found at other quantiles of the CIMT distribution. Another limitation is that the air pollution concentrations were only estimated at the participants’ residential addresses and do not account for the participants’ exposure outside their homes. However, the participants’ school exposure which has been estimated since the age of 5 years was correlated with residential exposure (r = 0.68–0.88 for primary school exposure and r = 0.36–0.73 for secondary school exposure) (Milanzi et al., 2018). Consequently, exposure misclassification arising from solely utilizing residential exposure is nondifferential and would likely lead to underestimated effects sizes of the air pollutants with wider confidence intervals.

While we found no clear patterns of association between PM_coarse_ and quantiles of CIMT, the PM_coarse_ exposure model’s leave one out cross-validation R^2^ was low at 38% (see Supplementary 1). In addition to a relatively small sample size, the low R^2^ could partially explain why we did not find an association or pattern between PM_coarse_ and CIMT. Overall, within the small range of air pollution exposures in our study, our findings are robust even after adjustment of multiple potential confounders and multiple testing with FDR.

## 6. Conclusion

Early childhood exposure to traffic-related air pollution was suggestively associated with an increase at 10th quantile of CIMT distribution during adolescence. Since CIMT increases with age, mitigation strategies to reduce traffic-related air pollution early in life could possibly delay atherosclerosis and subsequently CVD development later in life.

## Supplementary Material

Supplementary Materials

## Figures and Tables

**Table 1:** Characteristics of 363 adolescents study population with common carotid intima media thickness (CIMT) measurements in the PIAMA cohort.

Characteristics	Mean (SD)	N (%)
**Age**^a^ (years)	16.3 (0.2)	
**Sex**		
Male		183 (50.4)
Female		180 (49.6)
**Dutch nationality**		
Yes		334 (92.0)
No		29 (8.0)
**Body mass index**^a^ (kg/m^2^)	20.9 (2.7)	
**Total cholesterol**^a^ (mmol/1)	3.9 (0.7)	
**HDL cholesterol**^a^ (mmol/1)	1.4 (0.3)	
**Systolic blood pressure**^a^ (mm Hg)	115.9 (9.5)	
**Diastolic blood pressure**^a^ (mm Hg)	67.0 (6.8)	
**Maternal smoking during pregnancy**		
Yes		53 (14.6)
No		310 (85.4)
**Exposure to indoor smoking at birth**		
Yes		132 (36.4)
No		231 (63.6)
**Overweight mother before pregnancy**		
Yes		64 (17.6)
No		299 (82.4)
**Breastfeeding**		
No breastfeeding		45 (12.4)
<16 weeks		149 (41.0)
≥16 weeks		169 (46.6)
**Parental education**		
High		242 (66.7)
Low		121 (33.3)

a Measurement taken at the 16-year medical examination.

**Table 2:** Distribution of the estimated annual average air pollution concentrations at the birth address for nitrogen dioxide (NO_2_), fine particulate matter (PM_2.5_), PM_2.5_ absorbance, PM_coarse_ and ultrafine particles assigned at the participants’ residential birth addresses in the PIAMA cohort.

				Percentile									Spearnan’s conrrelation coefficients				
Variable	^n^observations	^n^missing	IQR	10th	20th	30th	40th	50th	60th	70th	80th	90th	NO_2_	PM_2.5_	PM_2.5_ absorbance	PM coarse	Ultrafine
NO_2_ (μg/m^3^)	363	2	5.31	19.28	21.40	22.70	23.79	24.55	25.69	26.77	27.83	29.69	1	0.40	0.78	0.73	0.81
PM_2.5_ (μg/m^3^)	363	2	0.43	16.43	16.49	16.52	16.57	16.65	16.71	16.89	17.03	17.20		1	0.68	0.62	0.37
PM_2.5_ absorbance (10^−5^m^−1^)	363	2	0.17	1.11	1.19	1.23	1.26	1.29	1.31	1.35	1.40	1.53			1	0.74	0.76
PM_coarse_ (μg/m^3^)	363	2	0.73	7.78	7.85	8.01	8.14	8.28	8.37	8.57	8.73	9.23				1	0.68
Ultrafine (paiticles/10,000 cm^3^)	363	2	0.15	0.96	1.00	1.03	1.07	1.10	1.13	1.16	1.19	1.27					1

Abbreviations: NO_2_– nitrogen dioxide; PM_2.5_− fine particulate matter; IQR- interquartile range.

**Table 3:** Changes in common carotid intima media thickness (CIMT) measured at age 16 years and exposure to air pollutants at birth for the PIAMA participants for different quantiles of the distributions of CIMT. The estimates and 95% bootstrap confidence intervals represent the change in CIMT (μm) for an IQR increase in the air pollution exposure adjusted for multiple testing with FDR. The IQR for each exposure is reported in the table.

Model I^a^ Quantile										
*Pollutant*	IQR	0.1	0.2	0.3	0.4	0.5	0.6	0.7	0.8	0.9
*NO*_*2*_	5.31	6.37 (−15.07, 27.01)	5.15 (−12.18, 22.48)	4.46 (−10.55, 19.47)	2.87 (−9.4, 15.14)	2.60 (−8.53, 13.74)	1.96 (−6.44, 10.37)	1.17 (−8.84, 11.17)	−1.01 (−9.65, 7.63)	6.80 (−30.12, 16.53)
*PM*_*2.5*_	0.43	4.78 (−0.07, 9.62)	3.24 (−1.46, 7.94)	3.09 (−1.39, 7.56)	2.23 (−2.2, 6.66)	1.23 (−2.07, 4.52)	0.57 (−3.65, 4.78)	0.02 (−4.13, 4.17)	−2.36 (−8.55, 3.83)	−3.39 (−9.84, 3.05)
*PM*_*2.5*_ *absorbancc*	0.17	**8.07 (2.13, 14.01)**	4.11 (−1.95, 10.16)	3.74 (−1.85, 9.33)	3.50 (−1.74, 8.74)	1.41 (−2.91, 5.73)	1.36 (−2.81, 5.52)	0.66 (−4.35, 5.68)	−2.73 (−11.11, 5.64)	−4.54 (−12.11, 3.03)
*PM coarsc*	0.73	1.00 (−236.32, 238.33)	1.94 (−457.12, 461)	−1.37 (−325.31, 322.57)	2.01 (−472.64, 476.66)	−0.03 (−6.96, 6.9)	0.40 (−93.15, 93.94)	−0.73 (−173.96, 172.5)	−4.57 (−1083.79, 1074.65)	−7.23 (−1715.28, 1700.82)
*Ultrafine particles*	0.15	5.69 (−19.00, 30.39)	3.07 (−10.24, 16.37)	1.26 (−15.03, 17.54)	1.02 (−12.16, 14.20)	0.14 (−3.68, 3.95)	0.45 (−5.40, 6.30)	−0.62 (−8.68, 7.44)	−1.92 (−26.78, 22.94)	−4.14 (−31.24, 22.96)
Model II^b^ Quantile										
*Pollutant*	IQR	0.1	0.2	0.3	0.4	0.5	0.6	0.7	0.8	0.9
*NO*_*2*_	5.31	7.11 (−17.10, 31.32)	5.58 (−13.4, 24.55)	3.24 (−30.04, 36.51)	1.12 (−25.06, 27.29)	1.06 (−23.87, 25.99)	0.9 (−20.29, 22.09)	0.32 (−7.16, 7.8)	0.74 (−18.2, 16.71)	−2.97 (−72.78, 66.84)
*PM*_*2.5*_	0.43	4.05 (−0.41, 10.10)	3.73 (−0.74, 8.20)	2.19 (−6.44, 10.83)	1.33 (−3.93, 6.60)	1.31 (−3.86, 6.49)	0.7 (−3.73, 5.13)	−0.4 (−4.82, 4.03)	−2.26 (−11.17, 6.65)	−1.82 (−8.98, 5.35)
*PM*_*2.5*_ *absorbance*	0.17	**6.23 (0.15, 12.3)**	5.03 (−2.67, 12.73)	3.04 (−4.03, 10.12)	1.81 (−6.01, 9.63)	1.69 (−5.59, 8.97)	1.06 (−4.66, 6.77)	−0.35 (−4.9, 4.2)	−2.07 (−11, 6.86)	−2.52 (−13.35, 8.32)
*PM coarse*	0.73	3.14 (−113.84, 120.12)	−0.61 (−23.29, 22.08)	−0.99 (−37.89, 35.91)	−2.18 (−83.35, 79)	−0.64 (−24.42, 23.14)	0.12 (−5.76, 6.01)	−1.20 (−45.75, 43.36)	−2.32 (−88.69, 84.05)	−2.08 (−79.42, 75.27)
*Ultrafine particles*	0.15	3.83 (−312.18, 319.85)	3.96 (−322.74, 330.66)	0.34 (−28.06, 28.75)	−0.62 (−51.4, 50.17)	0.10 (−8.44, 8.65)	−0.05 (−3.94, 3.85)	−0.96 (−79.90, 77.98)	−1.43 (−119.72, 116.85)	−3.13 (−261.2, 254.93)

a Adjusted for age, sex, parental education (high = vocational education or university studies), exposure to indoor tobacco smoke, maternal smoking status during pregnancy, maternal BMI (kg/m^2^), breastfeeding status and Dutch nationality (both parents born in the Netherlands).

b Adjusted for age, sex, parental education (high = vocational education or university studies), exposure to indoor tobacco smoke, maternal smoking status during pregnancy, maternal BMI (kg/m^2^), breastfeeding status, Dutch nationality (both parents born in the Netherlands), body mass index (BMI) (kg/m^2^), TC and HDLC (mmol/L) and mean systolic and diastolic pressure at the medical exam (mm Hg).
