# Supplementary material for: Quantile regression to examine the association of air pollution with subclinical atherosclerosis in an adolescent population
Source: Environ Int. Author manuscript; Available in PMC 2023 Feb 1. (PMC9890274; doi:10.1016/j.envint.2022.107285)
Supplement: Supplementary Materials [file NIHMS1868374-supplement-Supplementary_Materials.docx]

**Quantile regression to examine the association of air pollution with subclinical atherosclerosis in an adolescent population**

Adjani A. Peralta, Joel Schwartz, Diane R. Gold, Judith M. Vonk,
Roel Vermeulen, Ulrike Gehring

**Supplementary Material**

**Supplementary 1:** Land-use regression models with model R^2^.

| **Pollutant** | **Land-use regression model** | **R^2^** |
| --- | --- | --- |
| NO_2_ | -7.80 + 1.18×REGIONALESTIMATE + 2.30×10 ^- 5^×POP_5000 + 2.46×10 ^- 6^×TRAFLOAD_50 + 1.06×10 ^- 4^×ROADLENGTH_1000 + 9.84×10 ^- 5^×HEAVYTRAFLOAD_25 + 12.19×DISTINVNEARC1 + 4.47×10 ^- 7^×HEAVYTRAFLOAD_25_500 | 0.81^*^ |
| PM_2.5_ absorbance | 0.07 + 2.95×10^−9^×TRAFLOAD_500 + 2.93×10^−3^×MAJORROADLENGTH_50 + 0.85×REGIONALESTIMATE + 7.90×10^−9^×HLDRES_5000 + 1.72×10^−6^×HEAVYTRAFLOAD_50 | 0.89^*^ |
| PM_2.5_ | 9.46 + 0.42×REGIONALESTIMATE + 0.01×MAJORROADLENGTH_50 + 2.28×10^−9^×TRAFMAJORLOAD_1000 | 0.60^*^ |
| PM_10_ | 23.71 + 2.16×10 ^- 8^×TRAFMAJORLOAD_500 + 6.68×10 ^- 6^×POP_5000 + 0.02×MAJORROADLENGTH_50 | 0.61^*^ |
| PM_coarse_ | 7.59 + 5.02×10^−9^×TRAFLOAD_1000 + 1.38×10^−7^×PORT_5000 + 5.38×10 ^- 5^×TRAFNEAR | 0.38^*^ |
| Ultrafine particles | 7843+4688*HHOLD_5000+1499*HTRAFNEAR+4541*PORT_5000+3606*TLOA_50 + 2800*TMLOA_50+2881*TRAFNEAR | 0.51^§^ |

* =Leave one out cross-validation

§= External validation

DISTINVNEARC1: Inverse distance to the nearest road; HLDRES_X: Sum of high density and low density residential land in X m buffer; HEAVYTRAFLOAD_X: Total heavy-duty traffic load of all roads in a buffer (sum of (heavy-duty traffic intensity *length of all segments)); MAJORROADLENGTH_X; Road length of major roads in X m buffer; POP_X: Number of inhabitants in X m buffer; REGIONALESTIMATE: Regional estimate; ROADLENGTH_X: Road length of major roads in X m buffer; TRAFNEAR: Traffic intensity on nearest road; TRAFLOAD_X: Total traffic load of all roads in X m buffer (sum of (traffic intensity * length of all segments)); TRAFMAJORLOAD_X: Total traffic load of major roads in X m buffer (sum of (traffic intensity * length of all segments))

**Supplementary 2:** Comparison of characteristics between baseline population of the PIAMA cohort and the study population

|  | **Baseline population**  **N= 3963** | | **Study population**  **N= 363** | |
| --- | --- | --- | --- | --- |
| **Characteristics** | **n/N** | **%** | **n/N** | **%** |
| **Sex** |  |  |  |  |
| Male | 2054/3963 | 51.8 | 183/363 | 50.4 |
| Female | 1909/3963 | 48.2 | 180/363 | 49.6 |
| **Dutch nationality** |  |  |  |  |
| Yes | 3327/3684 | 90.3 | 334/363 | 92.0 |
| No | 357/3684 | 9.7 | 29/363 | 8.0 |
| **Smoking status during pregnancy** |  |  |  |  |
| Yes | 567/3918 | 14.5 | 53/363 | 14.6 |
| No | 3351/3918 | 85.5 | 310/363 | 85.4 |
| **Exposure to indoor smoking at birth** |  |  |  |  |
| Yes | 1729/3935 | 43.9 | 132/363 | 36.4 |
| No | 2206/3935 | 56.1 | 231/363 | 63.6 |
| **Overweight mother before pregnancy** |  |  |  |  |
| Yes | 686/3463 | 19.8 | 64/363 | 17.6 |
| No | 27777/3463 | 80.2 | 299/363 | 82.4 |
| **Breastfeeding** |  |  |  |  |
| No breastfeeding | 696/3896 | 17.9 | 45/363 | 12.4 |
| <16 weeks | 1934/3896 | 49.6 | 149/363 | 41.0 |
| ≥ 16 weeks | 1266/3896 | 32.5 | 169/363 | 46.6 |
| **Parental education** |  |  |  |  |
| High | 1908/3769 | 50.6 | 242/363 | 66.7 |
| Low | 1861/3769 | 49.4 | 121/363 | 33.3 |

**Supplementary 3:** Distribution of common carotid intima media thickness (CIMT) (μm) measured at age 16 years for the PIAMA participants.

**
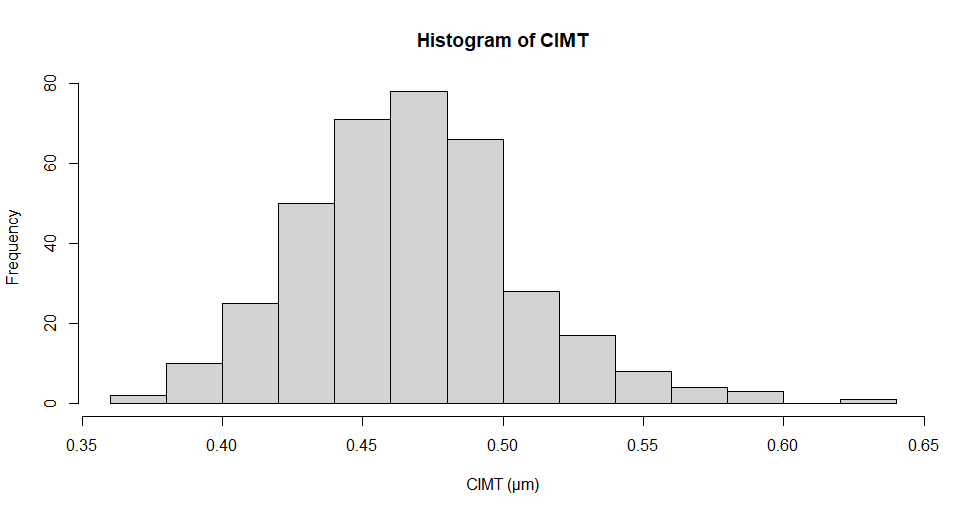
**

**Supplementary 4:** Distribution of the annual average air pollutant concentrations at the birth address for NO_2_, PM_2.5_, PM_2.5_ absorbance, PM_coarse_ and ultrafine particle sand CIMT within deciles of the CIMT distribution.

|  | **Decile of CIMT**  Mean (SD) | | | | | | | | | |
| --- | --- | --- | --- | --- | --- | --- | --- | --- | --- | --- |
| **Variable** | **1^st^** | **2^nd^** | **3^rd^** | **4^th^** | **5^th^** | **6^th^** | **7^th^** | **8^th^** | **9^th^** | **10^th^** |
| NO_2_  (μg/m³) | 23.09  (4.89) | 24.32  (3.85) | 24.79  (4.71) | 25.89  (4.05) | 26.25  (4.31) | 25.47  (4.68) | 23.48  (4.22) | 24.30  (4.17) | 24.88  (3.40) | 24.25  (4.03) |
| PM_2.5_  (μg/m³) | 16.49  (0.49) | 16.82  (0.47) | 16.71  (0.67) | 16.75  (0.33) | 16.98  (0.86) | 16.88  (0.63) | 16.85  (0.62) | 16.78  (0.42) | 16.79  (0.61) | 16.68  (0.61) |
| PM_2.5_ absorbance  (10^-5^m^-1^) | 1.24  (0.16) | 1.30  (0.15) | 1.30  (0.20) | 1.32  (0.15) | 1.40  (0.24) | 1.34  (0.21) | 1.32  (0.22) | 1.31  (0.13) | 1.30  (0.16) | 1.29  (0.18) |
| PM_coarse_  (μg/m³) | 8.29  (0.58) | 8.33  (0.55) | 8.41  (0.66) | 8.41  (0.52) | 8.58  (0.61) | 8.44  (0.75) | 8.31  (0.56) | 8.34  (0.56) | 8.25  (0.42) | 8.38  (0.49) |
| Ultrafine  (particles/10,000 cm^3^) | 1.09  (0.12) | 1.10  (0.13) | 1.12  (0.15) | 1.13  (0.11) | 1.17  (0.23) | 1.14  (0.20) | 1.10  (0.16) | 1.11  (0.14) | 1.09  (0.07) | 1.12  (0.12) |
| CIMT  (μm) | 405.49  (12.19) | 428.57  (3.61) | 441.32  (3.43) | 453.25  (3.66) | 462.03  (2.56) | 471.00  (3.49) | 481.14  (2.54) | 491.61  (3.26) | 504.67  (6.04) | 544.11  (23.90) |

**Supplementary 5:** Heatmap of Spearman correlations across between the five air pollutants (NO_2_, PM_2.5_, PM_2.5_ absorbance, PM _coarse_ and Ultrafine particles) and follow up periods.


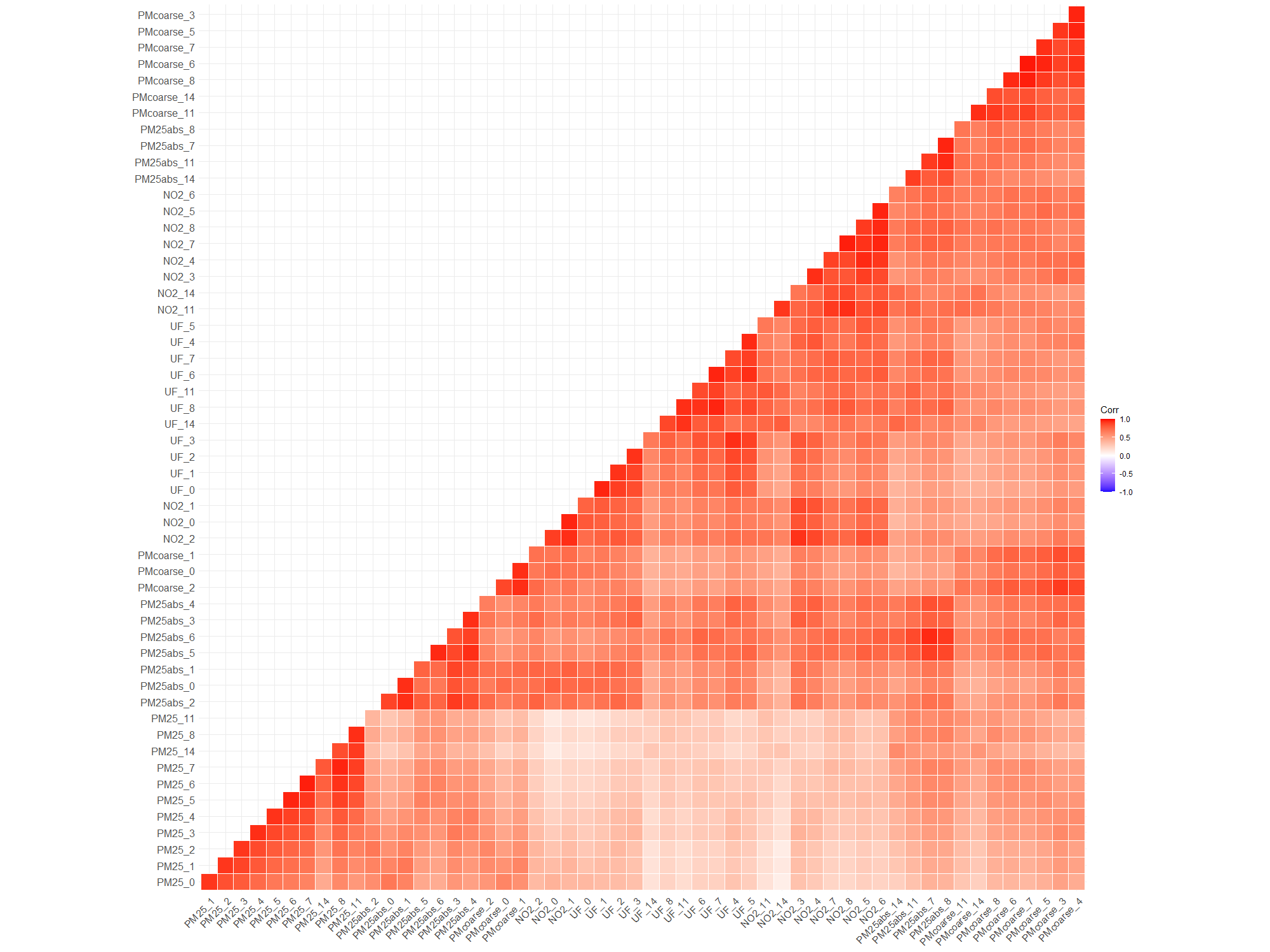


**Supplementary 6:** Differences in carotid intima media thickness (CIMT) measured at age 16 years reported for exposure to air pollutants up to the age of 14 years of the PIAMA participants for different quantiles of the distributions of CIMT (adjusted for age, sex, parental education, exposure to indoor tobacco smoke, maternal smoking status during pregnancy, maternal BMI (kg/m^2^), breastfeeding status, Dutch nationality, body mass index (BMI) (kg/m^2^), TC and HDLC (mmol/L) and mean systolic and diastolic pressure at the medical exam (mm Hg)). The estimates and 95% bootstrap confidence intervals represent the difference in CIMT (μm) for an IQR increment in the air pollution exposure adjusted for multiple testing with FDR. The IQR for each exposure is also reported in the table.

|  |  |  | **Quantile** | | | | | | | | | |
| --- | --- | --- | --- | --- | --- | --- | --- | --- | --- | --- | --- | --- |
|  |  |  | **0.1** | **0.2** | **0.3** | **0.4** | **0.5** | **0.6** | **0.7** | **0.8** | **0.9** |  |
| **Pollutant** | **Year** | **IQR** |  |  |  |  |  |  |  |  |  |  |
| NO_2_ | 0 | 5.31 | 7.11  (-17.10, 31.32) | 5.58  (-13.4, 24.55) | 3.24  (-30.04, 36.51) | 1.12  (-25.06, 27.29) | 1.06  (-23.87, 25.99) | 0.90  (-20.29, 22.09) | 0.32  (-7.16, 7.80) | -0.74  (-18.20, 16.71) | -2.97  (-72.78, 66.84) |  |
|  | 1 | 5.36 | 6.27  (-165.60, 178.14) | 4.23  (-1751.25, 1759.72) | 0.96  (-399.02, 400.95) | -0.59  (-245.02, 243.85) | 0.27  (-110.84, 111.37) | 0.00  (0.00, 0.00) | 2.68  (-1108.39, 1113.74) | 1.29  (-532.03, 534.60) | -2.73  (-1136.02, 1130.56) |  |
|  | 2 | 5.32 | 6.70  (-83.13, 96.53) | 2.07  (-33.70, 37.84) | 1.01  (-16.42, 18.44) | -1.28  (-23.29, 20.74) | -1.70  (-31.05, 27.65) | -0.05  (-7.06, 6.95) | -0.80  (-14.56, 12.96) | 1.33  (-21.60, 24.26) | -1.70  (-31.05, 27.65) |  |
|  | 3 | 5.27 | 3.11  (-277.13, 283.35) | 0.11  (-9.39, 9.61) | 0.16  (-14.09, 14.41) | -2.69  (-244.93, 239.56) | -2.58  (-235.32, 230.16) | -0.95  (-86.45, 84.55) | -0.90  (-81.64, 79.85) | 1.90  (-169.1, 172.89) | -1.74  (-158.48, 155.01) |  |
|  | 4 | 5.05 | -2.73  (-20.62, 15.17) | -2.53  (-19.10, 14.05) | -0.91  (-6.91, 5.10) | -3.38  (-25.59, 18.82) | -2.63  (-19.86, 14.61) | -2.73  (-20.62, 15.17) | -1.87  (-14.13, 10.39) | -4.65  (-35.14, 25.84) | -1.82  (-13.83, 10.19) |  |
|  | 5 | 5.23 | -2.46  (-11.46, 6.55) | -4.76  (-15.55, 6.04) | -4.60  (-11.73, 2.53) | -4.34  (-11.07, 2.39) | -6.12  (-15.60, 3.37) | -5.12  (-13.07, 2.82) | -2.88  (-13.32, 7.57) | -2.82  (-13.17, 7.53) | -5.49  (-23.20, 12.22) |  |
|  | 6 | 4.98 | -2.44  (-12.01, 7.13) | -3.58  (-16.57, 9.40) | -3.39  (-15.65, 8.88) | -3.39  (-15.65, 8.88) | -5.53  (-25.54, 14.49) | -3.88  (-17.95, 10.18) | -1.74  (-8.58, 5.09) | -3.68  (-18.14, 10.77) | -3.29  (-16.18, 9.60) |  |
|  | 7 | 4.93 | -4.88  (-21.39, 11.63) | -2.32  (-26.73, 22.09) | -1.97  (-22.74, 18.80) | -3.89  (-17.07, 9.28) | -4.53  (-19.88, 10.81) | -0.84  (-13.00, 11.33) | -1.53  (-19.54, 16.49) | -0.10  (-6.19, 5.99) | -6.06  (-26.57, 14.45) |  |
|  | 8 | 5.00 | -3.10  (-12.41, 6.20) | -3.65  (-13.83, 6.53) | -4.20  (-13.85, 5.44) | -4.55  (-15.00, 5.90) | -6.00  (-19.78, 7.77) | -4.40  (-14.51, 5.70) | -3.10  (-12.41, 6.20) | -2.75  (-11.01, 5.50) | -6.50  (-21.43, 8.42) |  |
|  | 11 | 4.85 | 0.58  (-8.44, 9.61) | -4.32  (-38.72, 30.08) | -3.11  (-27.85, 21.63) | -3.06  (-27.41, 21.29) | -3.69  (-33.07, 25.69) | -3.59  (-32.20, 25.01) | -1.65  (-27.22, 23.92) | -0.92  (-15.21, 13.37) | -0.92  (-15.21, 13.37) |  |
|  | 14 | 4.94 | -5.63  (-28.85, 17.59) | -3.60  (-18.48, 11.27) | -2.86  (-14.68, 8.95) | -3.60  (-18.48, 11.27) | -2.47  (-12.65, 7.72) | -3.65  (-18.73, 11.42) | -2.86  (-14.68, 8.95) | -1.48  (-8.97, 6.00) | 2.07  (-8.41, 12.55) |  |
| PM_2.5_ | 0 | 0.43 | 4.85  (-0.41, 10.10) | 3.73  (-0.74, 8.20) | 2.19  (-6.44, 10.83) | 1.33  (-3.93, 6.60) | 1.31  (-3.86, 6.49) | 0.70  (-3.73, 5.13) | -0.40  (-4.82, 4.03) | -2.26  (-11.17, 6.65) | -1.82  (-8.98, 5.35) |  |
|  | 1 | 0.44 | 4.34  (-3.31, 11.98) | 4.11  (-6.68, 14.89) | 2.09  (-46.15, 50.33) | 1.16  (-25.55, 27.86) | 0.31  (-6.90, 7.52) | 0.23  (-5.05, 5.51) | 0.42  (-9.33, 10.17) | -1.78  (-43.02, 39.45) | -1.52  (-36.76, 33.72) |  |
|  | 2 | 0.44 | 4.01  (-17.37, 25.40) | 3.15  (-61.32, 67.62) | 0.52  (-10.09, 11.13) | 0.64  (-12.40, 13.67) | 0.26  (-5.13, 5.66) | 0.91  (-17.70, 19.52) | 0.68  (-13.17, 14.52) | 0.45  (-8.72, 9.62) | -1.26  (-27.07, 24.55) |  |
|  | 3 | 0.43 | 3.06  (-143.47, 149.60) | 0.24  (-11.11, 11.59) | 0.29  (-13.54, 14.12) | 0.71  (-33.14, 34.55) | 0.18  (-8.28, 8.64) | 0.10  (-4.65, 4.85) | -0.20  (-9.69, 9.30) | 0.69  (-32.13, 33.50) | -1.15  (-56.05, 53.75) |  |
|  | 4 | 0.42 | 2.60  (-66.25, 71.46) | -1.30  (-35.55, 32.96) | -1.65  (-45.23, 41.93) | -0.39  (-10.72, 9.94) | 0.28  (-7.02, 7.58) | -0.55  (-15.04, 13.94) | -0.59  (-16.32, 15.13) | -1.15  (-31.59, 29.29) | -0.27  (-7.34, 6.81) |  |
|  |  |  | **Quantile** | | | | | | | | | |
|  |  |  | **0.1** | **0.2** | **0.3** | **0.4** | **0.5** | **0.6** | **0.7** | **0.8** | **0.9** |  |
| **Pollutant** | **Year** | **IQR** |  |  |  |  |  |  |  |  |  |  |
| PM_2.5_ | 5 | 0.42 | -2.46  (-14.23, 9.31) | -3.26  (-18.85, 12.32) | -4.22  (-16.29, 7.84) | -3.01  (-17.41, 11.38) | -1.45  (-8.39, 5.49) | -1.79  (-10.31, 6.74) | -1.56  (-8.99, 5.88) | 1.14  (-6.22, 8.49) | -2.13  (-12.31, 8.05) |  |
|  | 6 | 0.42 | -1.81  (-10.17, 6.55) | -3.58  (-20.06, 12.91) | -3.36  (-18.85, 12.13) | -2.62  (-14.69, 9.45) | -1.10  (-6.15, 3.96) | -2.00  (-11.20, 7.21) | -1.77  (-9.94, 6.40) | -2.15  (-12.07, 7.77) | -1.65  (-9.26, 5.96) |  |
|  | 7 | 0.41 | -3.58  (-69.22, 62.06) | -3.09  (-59.85, 53.67) | -1.93  (-37.37, 33.51) | -2.39  (-46.17, 41.4) | -1.02  (-19.68, 17.65) | -0.28  (-5.36, 4.81) | -0.93  (-18.08, 16.21) | -0.60  (-11.60, 10.40) | -1.63  (-31.53, 28.27) |  |
|  | 8 | 0.42 | 2.66  (-13.53, 18.85) | -1.99  (-14.11, 10.12) | -2.25  (-15.94, 11.44) | -1.88  (-13.34, 9.58) | -0.59  (-7.67, 6.50) | -0.33  (-4.30, 3.64) | -1.46  (-13.77, 10.86) | -0.80  (-10.44, 8.85) | -3.44  (-24.36, 17.48) |  |
|  | 11 | 0.42 | 0.89  (-16.20, 17.98) | -1.63  (-32.77, 29.52) | -3.11  (-62.60, 56.39) | -2.12  (-42.69, 38.45) | -0.75  (-15.04, 13.55) | -1.25  (-25.21, 22.71) | -0.25  (-5.04, 4.54) | 0.94  (-17.11, 18.99) | -0.90  (-18.07, 16.27) |  |
|  | 14 | 0.42 | -3.87  (-62.47, 54.74) | -3.00  (-48.55, 42.54) | -2.80  (-45.19, 39.60) | -2.28  (-36.92, 32.35) | -0.20  (-4.84, 4.44) | 0.17  (-3.80, 4.14) | -0.50  (-12.01, 11.02) | -1.33  (-32.19, 29.53) | -2.00  (-48.53, 44.53) |  |
| PM_2.5_  absorbance | 0 | 0.17 | 6.23  (0.15, 12.30) | 5.03  (-2.67, 12.73) | 3.04  (-4.03, 10.12) | 1.81  (-6.01, 9.63) | 1.69  (-5.59, 8.97) | 1.06  (-4.66, 6.77) | -0.35  (-4.90, 4.20) | -2.07  (-11.00, 6.86) | -2.52  (-13.35, 8.32) |  |
|  | 1 | 0.16 | 5.93  (-4.17, 16.03) | 3.52  (-15.38, 22.42) | 1.90  (-8.29, 12.08) | 0.91  (-3.98, 5.80) | 1.54  (-6.74, 9.82) | 1.18  (-5.18, 7.54) | 2.22  (-9.71, 14.15) | -1.38  (-8.82, 6.05) | -2.01  (-12.80, 8.78) |  |
|  | 2 | 0.16 | 5.50  (-7.45, 18.44) | 2.76  (-36.09, 41.61) | 0.71  (-9.33, 10.76) | 0.35  (-4.53, 5.22) | 0.51  (-6.69, 7.71) | 0.79  (-10.27, 11.84) | 0.63  (-8.24, 9.50) | 1.13  (-14.80, 17.06) | -1.33  (-20.05, 17.39) |  |
|  | 3 | 0.17 | 4.09  (-94.05, 102.23) | 0.47  (-10.75, 11.68) | 0.27  (-6.23, 6.77) | -1.67  (-41.61, 38.28) | 0.23  (-5.26, 5.71) | 0.66  (-15.15, 16.47) | -0.32  (-8.09, 7.44) | 1.14  (-26.25, 28.53) | -1.27  (-31.75, 29.21) |  |
|  | 4 | 0.16 | -0.45  (-8.23, 7.34) | -1.89  (-34.72, 30.95) | -0.75  (-13.77, 12.28) | -2.34  (-43.08, 38.4) | -2.12  (-38.93, 34.7) | -0.76  (-13.96, 12.44) | -1.07  (-19.77, 17.62) | -1.99  (-36.60, 32.62) | -0.48  (-8.75, 7.8) |  |
|  | 5 | 0.15 | -1.68  (-12.38, 9.01) | -3.30  (-16.85, 10.25) | -3.40  (-17.33, 10.54) | -4.25  (-21.71, 13.21) | -2.95  (-15.97, 10.06) | -1.12  (-8.23, 6.00) | -1.75  (-12.90, 9.40) | 1.27  (-6.83, 9.38) | -2.90  (-21.36, 15.56) |  |
|  | 6 | 0.15 | -1.02  (-10.63, 8.58) | -2.33  (-24.18, 19.52) | -3.12  (-32.41, 26.17) | -3.27  (-33.96, 27.42) | -1.93  (-20.05, 16.19) | -0.81  (-8.46, 6.83) | -1.36  (-14.17, 11.44) | -2.13  (-22.11, 17.85) | -1.02  (-10.56, 8.53) |  |
|  | 7 | 0.15 | -3.43  (-71.69, 64.84) | -2.29  (-47.80, 43.23) | -1.31  (-27.31, 24.70) | -2.72  (-56.94, 51.49) | -1.53  (-31.95, 28.90) | -0.25  (-5.28, 4.78) | -0.63  (-13.13, 11.88) | 0.99  (-18.67, 20.65) | -1.19  (-24.80, 22.43) |  |
|  | 8 | 0.15 | -2.53  (-14.01, 8.95) | -2.57  (-14.22, 9.09) | -3.29  (-18.21, 11.63) | -3.62  (-20.02, 12.79) | -1.90  (-10.51, 6.71) | -0.49  (-5.35, 4.37) | -2.23  (-12.36, 7.89) | -1.18  (-12.65, 10.29) | -2.15  (-19.10, 14.81) |  |
|  | 11 | 0.14 | -2.96  (-108.71, 102.78) | -2.67  (-97.89, 92.55) | -1.84  (-67.43, 63.75) | -2.01  (-73.58, 69.56) | -1.06  (-38.91, 36.79) | -0.75  (-27.62, 26.12) | -0.23  (-36.46, 36.00) | 1.16  (-40.17, 42.49) | 0.05  (-8.05, 8.15) |  |
|  | 14 | 0.14 | -3.78  (-94.63, 87.07) | -2.86  (-71.67, 65.95) | -2.39  (-59.76, 54.98) | -0.24  (-6.05, 5.56) | -0.23  (-5.76, 5.30) | 0.16  (-3.62, 3.94) | -0.62  (-15.54, 14.30) | -1.58  (-39.48, 36.32) | 0.33  (-7.50, 8.16) |  |
| PM coarse | 0 | 0.73 | 3.14  (-113.84, 120.12) | -0.61  (-23.29, 22.08) | -0.99  (-37.89, 35.91) | -2.18  (-83.35, 79.00) | -0.64  (-24.42, 23.14) | 0.12  (-5.76, 6.01) | -1.20  (-45.75, 43.36) | -2.32  (-88.69, 84.05) | -2.08  (-79.42, 75.27) |  |
|  | 1 | 0.76 | 0.00  (0.00, 0.00) | -0.20  (-2370.42, 2370.02) | -1.96  (-40.62, 36.71) | -1.99  (-41.41, 37.42) | -1.29  (-26.76, 24.19) | -1.17  (-24.40, 22.05) | -1.97  (-40.93, 36.99) | -4.17  (-86.59, 78.25) | -2.00  (-41.56, 37.56) |  |
|  | 2 | 0.76 | 1.80  (-26.95, 30.54) | -0.50  (-8.47, 7.47) | -2.40  (-40.68, 35.89) | -2.19  (-37.22, 32.83) | -1.38  (-23.36, 20.61) | -0.39  (-6.67, 5.89) | -1.17  (-19.89, 17.55) | -2.40  (-40.81, 36.00) | -1.64  (-27.85, 24.57) |  |

|  |  |  | **Quantile** | | | | | | | | | |
| --- | --- | --- | --- | --- | --- | --- | --- | --- | --- | --- | --- | --- |
|  |  |  | **0.1** | **0.2** | **0.3** | **0.4** | **0.5** | **0.6** | **0.7** | **0.8** | **0.9** |  |
| **Pollutant** | **Year** | **IQR** |  |  |  |  |  |  |  |  |  |  |
| PM coarse | 3 | 0.77 | -4.48  (-25.32, 16.37) | -2.63  (-17.6, 12.34) | -4.18  (-23.63, 15.28) | -3.73  (-21.12, 13.65) | -3.40  (-19.21, 12.42) | -2.61  (-17.49, 12.27) | -1.76  (-13.58, 10.07) | -1.33  (-10.26, 7.61) | -2.27  (-17.56, 13.02) |  |
|  | 4 | 0.77 | -5.79  (-19.96, 8.37) | -4.43  (-15.27, 6.40) | -4.62  (-15.92, 6.68) | -4.55  (-15.69, 6.58) | -3.48  (-13.90, 6.94) | -3.55  (-15.77, 8.67) | -1.70  (-14.12, 10.72) | -2.50  (-18.57, 13.56) | 0.22  (-11.00, 11.45) |  |
|  | 5 | 0.76 | -2.22  (-14.16, 9.71) | -3.97  (-12.24, 4.30) | -3.98  (-12.28, 4.32) | -5.70  (-16.71, 5.30) | -6.56  (-19.21, 6.10) | -5.11  (-15.76, 5.54) | -3.13  (-19.93, 13.67) | 1.63  (-14.29, 17.55) | -1.26  (-13.53, 11.02) |  |
|  | 6 | 0.75 | -1.64  (-10.81, 7.54) | -2.50  (-16.22, 11.22) | -3.86  (-24.99, 17.27) | -4.33  (-28.04, 19.38) | -2.85  (-18.45, 12.76) | -4.34  (-28.09, 19.42) | -1.79  (-11.85, 8.26) | -3.93  (-25.48, 17.61) | -1.85  (-12.20, 8.50) |  |
|  | 7 | 0.74 | -5.63  (-37.00, 25.75) | -2.20  (-14.45, 10.06) | -2.09  (-13.77, 9.58) | -4.33  (-28.46, 19.81) | -2.35  (-15.48, 10.77) | -3.22  (-21.19, 14.74) | -1.73  (-11.37, 7.92) | 2.58  (-11.82, 16.99) | 2.12  (-9.72, 13.96) |  |
|  | 8 | 0.74 | -2.99  (-17.82, 11.83) | -1.96  (-11.66, 7.74) | -2.24  (-13.33, 8.85) | -3.72  (-22.13, 14.70) | -2.54  (-15.14, 10.05) | -3.87  (-23.06, 15.31) | -3.22  (-19.14, 12.71) | 2.85  (-11.28, 16.99) | 0.17  (-10.61, 10.95) |  |
|  | 11 | 0.75 | -3.73  (-162.55, 155.10) | -2.34  (-102.16, 97.48) | -0.19  (-8.16, 7.79) | -1.32  (-57.45, 54.81) | -1.77  (-77.03, 73.50) | -3.86  (-168.42, 160.70) | -1.83  (-79.64, 75.99) | 0.37  (-15.57, 16.32) | -1.27  (-55.49, 52.94) |  |
|  | 14 | 0.72 | -5.65  (-23.59, 12.28) | -6.09  (-25.42, 13.24) | -3.98  (-16.61, 8.65) | -3.58  (-14.93, 7.77) | -1.26  (-9.29, 6.77) | -3.19  (-13.31, 6.93) | -3.76  (-15.68, 8.16) | -3.05  (-22.13, 16.04) | -1.72  (-12.74, 9.29) |  |
| Ultrafine | 0 | 0.15 | 3.83  (-312.18, 319.85) | 3.96  (-322.74, 330.66) | 0.34  (-28.06, 28.75) | -0.62  (-51.40, 50.17) | 0.10  (-8.44, 8.65) | -0.05  (-3.94, 3.85) | -0.96  (-79.90, 77.98) | -1.43  (-119.72, 116.85) | -3.13  (-261.20, 254.93) |  |
|  | 1 | 0.15 | 4.56  (-77.09, 86.21) | 2.42  (-40.86, 45.69) | 0.39  (-6.54, 7.31) | -1.49  (-28.22, 25.24) | -1.27  (-23.96, 21.42) | -0.25  (-4.69, 4.20) | -0.76  (-14.45, 12.92) | -1.29  (-24.47, 21.88) | -2.38  (-44.95, 40.20) |  |
|  | 2 | 0.15 | 4.41  (-89.47, 98.30) | 1.62  (-32.92, 36.17) | 0.28  (-5.64, 6.20) | -1.46  (-32.54, 29.62) | -1.59  (-35.39, 32.21) | -0.24  (-5.32, 4.84) | -0.70  (-15.64, 14.23) | -0.40  (-8.86, 8.06) | 2.01  (-40.81, 44.83) |  |
|  | 3 | 0.14 | 2.77  (-95.19, 100.74) | -0.29  (-10.40, 9.83) | -1.59  (-57.59, 54.42) | -2.50  (-90.95, 85.94) | -2.44  (-88.80, 83.91) | -0.40  (-14.60, 13.80) | -0.81  (-29.41, 27.79) | -0.22  (-7.89, 7.46) | 1.24  (-42.61, 45.09) |  |
|  | 4 | 0.14 | -4.05  (-39.92, 31.81) | -1.59  (-21.86, 18.68) | -2.26  (-22.21, 17.70) | -2.71  (-26.64, 21.23) | -5.55  (-19.48, 8.38) | -2.63  (-25.89, 20.63) | -1.21  (-16.61, 14.19) | -0.12  (-8.55, 8.32) | 1.46  (-17.17, 20.10) |  |
|  | 5 | 0.13 | -4.92  (-17.52, 7.67) | -3.48  (-12.19, 5.23) | -4.16  (-12.04, 3.71) | -3.79  (-10.95, 3.37) | -6.48  (-16.21, 3.26) | -4.45  (-12.85, 3.96) | -2.20  (-14.04, 9.64) | -1.04  (-10.45, 8.38) | 1.06  (-8.58, 10.71) |  |
|  | 6 | 0.13 | -5.43  (-26.18, 15.32) | -2.97  (-14.29, 8.36) | -2.14  (-10.32, 6.04) | -3.31  (-15.95, 9.33) | -3.95  (-19.03, 11.13) | -2.18  (-11.28, 6.92) | -1.15  (-8.69, 6.39) | 4.15  (-11.69, 19.99) | 2.48  (-10.48, 15.43) |  |
|  | 7 | 0.13 | -5.70  (-23.03, 11.63) | -2.65  (-16.90, 11.59) | -2.77  (-11.19, 5.65) | -3.24  (-13.11, 6.63) | -5.40  (-18.76, 7.96) | -3.62  (-14.63, 7.39) | -1.48  (-10.71, 7.74) | -0.62  (-9.54, 8.30) | -2.25  (-16.21, 11.72) |  |
|  | 8 | 0.13 | -5.64  (-20.09, 8.80) | -3.00  (-12.33, 6.32) | -3.20  (-11.43, 5.03) | -3.55  (-12.65, 5.54) | -5.62  (-16.83, 5.60) | -3.85 (-13.69, 6.00) | -2.27  (-9.32, 4.78) | -2.87(-11.79, 6.04) | -3.33(-13.65, 6.99) |  |
|  | 11 | 0.13 | -5.42  (-37.71, 26.87) | -2.32  (-16.12, 11.48) | -1.78  (-12.40, 8.83) | -2.34  (-16.29, 11.61) | -2.65  (-18.44, 13.14) | -1.81  (-12.59, 8.97) | -1.78  (-12.41, 8.84) | -0.64  (-9.04, 7.76) | 1.11  (-13.50, 15.72) |  |
|  | 14 | 0.13 | -6.35  (-36.02, 23.33) | -4.40  (-24.97, 16.17) | -2.06  (-11.67, 7.56) | -2.16  (-12.27, 7.94) | -1.96  (-11.12, 7.20) | -2.32  (-13.18, 8.54) | -1.91  (-10.82, 7.01) | -0.68  (-8.59, 7.22) | 1.12  (-11.82, 14.06) |  |
